# A New Interpretation of Relative Importance on an Analysis of Per and Polyfluorinated Alkyl Substances (PFAS) Exposures on Bone Mineral Density

**DOI:** 10.3390/ijerph20054539

**Published:** 2023-03-03

**Authors:** Andrea B. Kirk, Alisa DeStefano, Alexander Martin, Karli C. Kirk, Clyde F. Martin

**Affiliations:** 1 U.S. Environmental Protection Agency, Washington, DC 20460, USA; 2 Department of Mathematics and Computer Science, College of the Holy Cross, Worcester, MA 01610, USA; 3 Department of Computer Science, University of Rochester, Rochester, NY 14627, USA; 4 Department of Computer Science, University of Texas at Austin, Austin, TX 78712, USA; 5 Department of Mathematics and Statistics, Texas Tech University, Lubbock, TX 79409, USA

**Keywords:** relative importance analysis, vector space methods, PFAS, bone mineral density, gender, male, female

## Abstract

Background: The relative contribution of environmental contaminants is an important, and frequently unanswered, question in human or ecological risk assessments. This interpretation of relative importance allows determination of the overall effect of a set of variables relative to other variables on an adverse health outcome. There are no underlying assumptions of independence of variables. The tool developed and used here is specifically designed for studying the effects of mixtures of chemicals on a particular function of the human body. Methods: We apply the approach to estimate the contributions of total exposure to six PFAS (perfluorodecanoic acid, perfluorohexane sulfonic acid, 2-(N-methyl-PFOSA) acetate, perfluorononanoic acid, perfluoroundecanoic acid and perfluoroundecanoic acid) to loss of bone mineral density relative to other factors related to risk of osteoporosis and bone fracture, using data from subjects who participated in the US National Health Examination and Nutrition Surveys (NHANES) of 2013–2014. Results: PFAS exposures contribute to bone mineral density changes relative to the following variables: age, weight, height, vitamin D2 and D3, gender, race, sex hormone binding globulin, testosterone, and estradiol. Conclusion: We note significant alterations to bone mineral density among more highly exposed adults and significant differences in effects between men and women.

## 1. Introduction

Relative importance analysis allows for calculation of the importance of the contribution of one or more variables to an outcome of interest. Relative importance analysis has been used in such diverse applications as evaluations of contributions of body composition and fat distribution variables on blood pressure [1], the influence of genetics, self-identified ethnicity and socioeconomic status on serum creatinine levels [2] and personality factors leading to protection from, or vulnerability to, post traumatic stress disorder in soldiers [3]. Relative importance analysis has not been commonly used to evaluate the effects of environmental contaminants on health outcomes in humans. However, the ability to account for the toxicities of chemical mixtures in populations subject to multiple stressors is becoming increasingly important as the US EPA, the European Union and other regulatory agencies begin to undertake more complex risk assessments [4,5]. The consideration of mixtures, environmental justice issues, and the complexities of sub-population vulnerabilities in such assessments will require new tools and strategies. In this paper, a new tool is developed that allows a more effective attack on the problem of determining how much comparative effect an individual adverse actor has on a multi-factorial risk profile among free-living human subjects. This tool improves upon other applications of relative importance analysis and is rooted in the theory of optimization by vector space methods. It will allow us to understand the relative effect of a set of PFAS chemicals compared to a class of known influences. It does not allow us to compare the individual induced risk. The main goal of the paper is to understand this relative risk. To this end, the first part of the paper is devoted to the analysis of the risk and the second part of the paper will develop this statistical tool. The two parts of the paper can be read separately.

It is important to understand how much risk a particular chemical contributes to an adverse outcome compared to other factors such as co-exposures to other environmental contaminants, drugs, nutritional inadequacies, and disease states so that risks from individual environmental chemical exposures can be adequately addressed. For this model, we examine the contributions of the common environmental contaminant class per- and polyfluorinated alkyl substances (PFAS) on bone mineral density (BMD). We have chosen this pair because PFAS exposures are near ubiquitous among the US population, PFAS have been documented to adversely impact bone, and there are described mechanisms by which PFAS exposures interfere with bone homeostasis  [6].

A number of studies have evaluated the effects of PFAS on bone. These include in vitro and in vivo studies [7], epidemiology [8,9,10] and ex vivo human cadaver studies [7]. As incidence of fractures [11] and total numbers of fractures increase [12] with population age [13], this burden will also increase. Within the US, estimated costs for osteoporosis and fracture among the elderly exceeded 22 billion dollars for 2008 [14,15]. Hip fracture can be an especially devastating injury. More than 20% of people suffering acetabular fractures die within a year [16]. While this paper is concerned with increased fracture risk in PFAS-exposed adults, it will be important to remember that PFAS exposures are lifelong. The fetal skeletal system may also be affected by PFAS as a vast majority of US infants are exposed prenatally and through infancy [17]. This may predispose generations of children to osteoporosis or other bone-related health problems later in life.

### 1.1. What Are PFAS?

PFAS are a large class of fluorinated organic compounds that have been and are widely used in many types of industrial processes and products. Among these uses are aqueous fire-fighting foams (FFF), waterproof and/or breathable coatings for fabrics, and stain resistance treatments for clothing, carpeting, upholstery, etc. For a detailed review, see [18,19]. PFAS have found their way into drinking water from use of FFF at airports, military installations, and firefighting training activities. Other sources of PFAS exposure include leachate from landfills, emissions or releases from manufacturing sites and the use of PFAS-contaminated sludge in agriculture. Serum PFAS concentrations are also associated with the consumption of fast and prepackaged foods, diet and use of personal care products [20]. Their volatile properties, which vary by chemical, and tendency to present in dust, mean inhalation exposures and atmospheric transport occur [21]. PFAS have been found in some of the most remote areas of the globe [22]. They are commonly detected in serum samples collected from individuals in North America, Europe [23], Asia [24], and Africa [25].

PFAS have a wide variety of chemical structures, they interact with a number of different receptors and are associated with a wide array of adverse biological effects. Potential health impacts include metabolic and cardiovascular diseases, cancer, reduced immune function and altered bone development and homeostasis. For this exercise, we use BMD, which is an endpoint that is known to be influenced by a number of non-toxicological variables. We evaluate the importance of PFAS on BMD relative to other factors known to influence BMD, osteoporosis and fracture risk.

### 1.2. Why Relative Importance Analysis?

Relative importance analysis is a method used to evaluate the relative weight of individual predictors in regression equations and can be used to identify or estimate relative effect size [26]. Applying relative importance analysis to problems of chemical risk assessment can facilitate regulatory decisions for chemical mixtures. Mixtures present common and well-recognized regulatory challenges, yet chemicals remain assessed primarily as single, independent risks. Similarly, toxicological endpoints may have multiple contributors, and it can be difficult, particularly in epidemiology, to determine the contribution a chemical or chemical mixture makes to a disease state in a free-living population. In this paper, we diverge from the classical relative importance theory because we are dealing with a mixture of PFAS chemicals, and their relationship to bone mineral density is not clear. Almost every person who tested positive for any one PFAS then tested positive for multiple species of PFAS. Testing for the contribution of a given PFAS to bone density is then confounded by the fact that we do not know the relationship of that PFAS to others in the human system. They are almost surely not independent. Thus, we treat the PFAS as a group and use the methods developed in this paper to try to understand group effect. This idea of treating mixtures by vector space methods is being analyzed in great detail in an upcoming publication.

### 1.3. Goals of this Paper

Relative risks are best studied using large data sets collected from studies of the general population such as those provided by the US National Health and Nutrition Examination Surveys (NHANES) [27]. The NHANES effort has been underway for nearly 25 years and consists of self-reported survey data, physical examination and laboratory data for about 10,000 persons per study, cross-sectional, national representation of the US population. Data from the 2013–2014 surveys [27] will be used in this paper to demonstrate risks to BMD posed by PFAS

The main statistical goal of this paper is to develop a tool that will allow a more effective attack on the problem of determining how much comparative effect a variable has on an outcome detrimental to human health. For example, when evaluating a disease outcome, such as osteoporosis or bone fracture, it is important to know how much damage a particular chemical does to bone as compared to other factors. The problem is that many things affect bone strength and quality such as age, weight, physical activity, nutritional status, pharmaceuticals, cancer and a myriad of other factors.

In Section 3, we first give an example using data from the 2013–2014 NHANES data, and we then give a detailed analysis of the algorithm to produce the relative importance data.

In Section 4, we analyze the relative importance data obtained from applying our method to 2013–2014 NHANES data. In particular, we analyze the information contained in the first table from Section 3. We then give some thoughts on where the tool developed in this paper might be applied. We conclude this part of the paper with a summary of the toxicological aspects of the paper. In Section 4, we sketch the derivation of the main statistical results of the paper.

Appendix A contains pseudo-codes for the QR factorization and the modified Boot Strap used in our analysis.

## 2. Methods

We were interested in the effect of the PFAS chemicals on bone minearal density (BMD). Other BMD-influencing variables selected were Age (A), Albumin (AL) [28], Weight (W), Height (H), Testosterone (T), Estradiol (E), Vitamin D2 (D2), Vitamin D3 (D3), Epi-25OHD3 (ED3), Gender (G), Race (R), Potassium (P), and Sex Hormone Binding Globulin (SHBG(S)) along with six PFASs: perfluorodecanoic acid (PFDA), perfluorohexane sulfonic acid (PFHxS), 2-(N-Methyl-perfluorooctane sulfonamido) acetic acid(Me-PFOSA-AcOH), perfluorononanoic acid (PFNA), perfluoroundecanoic acid (PFUA) and n-perfluorooctane sulfonic acid (n-PFOS). These PFAS were selected because there were data available in a range of serum concentrations among subjects and because large numbers of subjects had detectable concentrations in their serum (Table 1), rather than out of concern that these specific PFAS impact BMD.

In Table 2, the first column is the common acronym for the particular PFAS, the second column is the NHANES identifier for the particular PFAS, the third column is the number of subjects that were tested for that particular PFAS, the fourth column is the number of subjects that tested below the minimal level of testing, and the last column is the percentage of subjects that tested above the minimal of detection.

Therefore, we did not evaluate relative contributions of some of the most well-known PFAS such as PFOA or focus on chemical structures (i.e., we did not choose to compare branched vs. linear PFAS) for this application. The purpose of this excercise is to demonstrate how this new tool can be used to evaluate the impact of an environmental contaminant on a health effect under the influence of other known factors.

We want to know the relative effect of these six PFAS chemicals as compared to the effect of Age, Albumin, Weight, Height, Testosterone, Vitamin D2, Vitamin D3, Epi-25OHD3, Race, Gender, SBGH and Estradiol on trunk bone mineral density (TBMD). “Trunk bone” is comprised of thoracic and lumbar spine, left and right ribs, and pelvis. To measure this, we apply the algorithm that is described in detail in Section 3.2. The mathematical and statistical derivation of the algorithm is contained in Section 4.

### Relative Importance Analysis: Background

A very old problem in the application of statistics is to rank effects. In [29], there is a very clever example involving a restaurant in which one wants to know what influences a customer to recommend the restaurant to friends. In this example, data were collected on Value, Service, and Food Quality, as well as data on whether or not a customer would recommend the restaurant. In this scenario, a ranking of the three variables was desired. In the two review papers  [26,30], the goal is much the same, that is, to rank individually a set of qualities (The fifth author would like to acknowledge Professor Jingyong Su for showing him this literature). Our goal is different in that we want to understand the effect of a set of chemicals on a toxicological endpoint that is also subject to other forces. We do not address the relative importance of individual chemicals.

The primary statistical tool used in Relative Importance Analysis is multiple linear regression. If we used MLR for our problem, we would find a linear function of 19 variables that would tell us how well TBMD is approximated by the 19 variables–the known quantities that affect TBMD–and the six species of PFAS. We are not interested in the individual quantities but only in how the class of PFAS affects TBMD as compared to the known quantities. In order to do this, we construct the vector space that corresponds to the known quantities and the vector space that corresponds to the known quantities as well as the PFAS group. We then calculate how well TBMD is approximated in the space of known quantities by taking as a measure of the approximation the length of the projection of the vector onto that space. Then, we do the same for the total space. In essence, we are calculating linear regression but on subspaces rather than individual vectors.

## 3. Applications

In this section, we use the method developed in the previous section to analyze the effect of PFAS on BMD. We first consider the effect of PFAS on a single target, trunk bone mineral density (TBMD). We then briefly discuss the algorithms used in the analysis. We then consider the effect of PFAS on multiple targets involving trunk bone mineral density along with other sites in the bone structure of interest.

### 3.1. Relative Importance of a Set of Data

In the 2013–2014 NHANES data, a set of subjects were tested for nine species of PFAS of which six had occurred at or above measurable levels (above 0.07 ng/mL) in at least 40% of the subjects. In Table 2, we give the justification for the choice of PFAS species. We wanted to know what effect the six PFAS species had on TBMD as a collection rather than individually. We want to know the relative effect of these six PFAS chemicals Perfluorodecanoic acid = LBXPFDE = PFDA, perfluorohexane sulfonic acid = LBXPFHS = PFHxS, 2-(N-Methyl-perfluorooctane sulfonamido) acetic acid= LBXMPAH = Me-PFOSA-AcOH, perfluorononanoic acid = LBXPFNA = PFNA, perfluoroundecanoic acid = LBXPFUA = PFUA and n-perfluorooctane sulfonic acid (n-PFOS) = LBXMFOS = PFOS. For the convenience of the reader, these are listed in Table 3 and Table 4. Trunk Bone Mineral Density (TBMD) was chosen as a surrogate for BMD in general.

We selected those subjects that were tested for all 20 variables (1004 subjects). We then created two new files—one for male subjects and one for female subjects. There were 528 female subjects and 476 male subjects selected. The data were normalized by applying the transformation given by

$$T\left(x_{ij}\right)=\frac{x_{ij}-E\left(X_{i}\right)}{\sigma (X_{i})}$$

where *E* is the mean or average, σ is standard deviations and $T(x_{ij})$ is the new data point. Based on the initial data set for combined males and females, we generated 1002 random data sets by randomly removing two subjects from the data set. Then, for each of these data sets, we formed two matrices from the data. The first data set consisted of the data from the set

A={A,AL,G,D2,D3,E,ED3,H,P,R,T,W,PFDA,PFxS,Me−PFOSA−AcOH,PFNA,PFUA,n−PFOS}

and the second was the data from the set

B={A,AL,G,D2,D3,E,ED3,H,P,R,T,W}.

For the combined data, we considered each datum to be a vector of length 1004, and the set then generates a subspace of $R^{1004}$. The subspace is of dimension 19 for A and 13 for B. We then construct an orthonormal basis by applying the QR algorithm to the matrix corresponding to the original data. That is, we factor A=(QA)∗(RA) and B=(QB)∗(RB), and then QA and QB are orthonormal bases for the two spaces. We used the Householder QR algortihm for this calculation. Now, the data from TBMD is also a vector in the large vector space. Our goal is to approximate TBMD in the two spaces and to compare the level of approximation. To this end, we calculated

$$a=QA*QA^{\prime }*TBMD$$

and

$$b=QB*QB^{\prime }*TBMD.$$

We calculated the percentage change in length,

$$e=\frac{a-b}{b}.$$

We did this for all 1002 data sets using the program “Modified Bootstrap” (see Appendix A). We then performed similar calculations for both the male and female subjects. We report the statistics for the male, female and combined data sets in Table 5. The addition of PFAS causes an 11% increase in the approximation for the female subjects.

### 3.2. The Algorithm

In this section, we describe the total algorithm used.

**Step 1:** Collect the data in tabular form using either Excel or Matlab. In tabular form, it needs to have many more rows than columns. The method is not applicable to data sets with fewer subjects than categories. In this paper, arrays were typically approximately 600 rows and 30 or fewer columns.

**Step 2:** Adjust for missing data. Some software programs have built-in capability to resolve the problem, but if there are massive amounts of missing data, the data set must be modified. The data set used in this paper contains data collected from about 10,000 subjects with the data in several hundred categories. However, not every subject was tested in every category. For this study, we selected only those subjects that had been tested in each of the selected categories. Go to **Step 3**.

**Step 2b:** Remove two randomly selected subjects from the data [31]. Go to **Step 3**.

**Step 3:** Divide the data set into three sets. The first, *X*, is the set of basic data. The second, *Y*, is the set for which the relative impact is needed. The third, *Z*, is the set by which the relative importance will be measured. The first two must have at least one column and Z has exactly one column.

**Step 4:** Calculate the mean and standard deviation of each column and record. Transform each table by using the transform

$$T\left(x_{ij}\right)=\frac{x_{ij}-E\left(X_{i}\right)}{\sigma (X_{i})}.$$

**Step 5:** Upload the transformed data, matrices XY, *X* and *Z* to MatLab.

**Step 6:** Factor the matrices XY and *X* using one of the QR algorithms. Denote the *Q* matrices as $Q_{XY}$ and $Q_{X}.$

**Step 7:** In Matlab, construct $A=Q_{XY}*Q_{XY}^{\prime }*Z$ and $B=Q_{X}*Q_{X}^{\prime }*Z$.

**Step 8:** Calculate the length of *A* and of *B*. Denote the lengths as *a* and *b*. This can be completed in Matlab or in Excel.

**Step 9:** Calculate the percentage change as δ=(a−b)/b.

**Step 10:** Record a, b and δ. Go to **Step 2b**.

**Step 11:** Calculated the average of the stored δs.

We constructed two vector spaces as surrogates for the information contained in the described variables. The space was spanned by the columns of Matrix1 and the space spanned by the columns of Matrix2. We then approximated the column of Matrix3 in each of the two spaces. This approximation is a surrogate for the amount of information about Matrix3 that is contained in the two matrices. The lengths of the columns were then compared to give the percent change.

## 4. Discussion

We have found that the bones of women are much more strongly affected by this group of PFAS than the bones of men (Table 5). The percentage increase of effect for women is 3.4 times the percentage increase for men. This is a potentially highly significant finding, although gender-specific differences in cholesterol, liver, ADHD associated with PFAS exposure have been noted [32,33]. If gender is not considered in studies of effects of PFAS on bone, significance for women and girls may be obscured. We strongly urge that all future studies of PFAS effects consider gender and report statistical, or clinical, significance for both sexes separately. We urge further investigation of the effects of PFAS on bone mineral density, particularly among women, as they are already more likely to lose bone mass, particularly with age, and they are already at higher risk of osteoporosis and fracture. This analytical approach does not allow for determination of direction of effect. So, this study does not say that PFAS decreases or increases BMD. However, others report associations between PFAS exposures and reduced BMD in female adolescents but not in males [34], and with reduced BMD and increased risk of osteoporosis among women [35].

Secondly, for a new approach to chemical risk assessments: One possible use for this type of risk analysis is a more robust analysis of total risk of environmental contaminants to public and environmental health. Rather than identifying the most sensitive endpoint from an animal study, say cancer or impaired neurodevelopment, and assuming that all other endpoints would be avoided by setting an exposure standard protective of that most sensitive endpoint, it might be possible, and potentially more protective of public health to estimate total risk/costs posed by a chemical: cancer and impaired neurodevelopment and loss of life due to heart disease. This would be especially beneficial when assessing risks posed by chemicals, or chemical classes, with multiple targets or adverse outcomes [36]. Such an approach would be complementary to the usual cumulative risk assessments where the total risk posed by a series of chemicals with a common target or outcome is evaluated.

The data we used is cross-sectional and represents a snapshot of subject exposures, health and physical vulnerabilities. Such data cannot be used to make causal inference. We propose further investigation of the potential for cumulative risk assessments of single chemicals, or mixtures, such as PFAS, on the total impact of multiple pathways disruptions and outcomes.

## 5. Mathematics and Statistics

In the following sections, we develop the tools to conduct the analysis of the previous sections. Here, we keep the biology to a minimum and give a concise development of this new approach to relative importance analysis. We treat relative importance analysis as a subtopic of information theory [37,38] and reduce the construction to a vector space calculation. We provide numerically stable tools to complete the construction.

A standard tool for attacking problems of relative risk has been through the use of linear regression. This paper reexamines regression along with relative importance analysis and shows that there is a way to attack the problem using the theory of linear vector spaces. The method is based on the material in [39] and was used, for example, in a development of control theoretic and statistical smoothing splines in [40]. In order to use vector space methods effectively, it is necessary to use material from numerical linear algebra, and the methods used here depend heavily on the work in [41,42]. The use of numerically stable algorithms is required in order to preserve numerical accuracy. The main thrust of this work is the reduction of a basis of a vector space to an orthonormal basis, i.e., various forms of the QR algorithm.

We review the basic construction of linear regression, although any basic applied statistics book will contain this material, for example [43]. Both the construction of the linear system for regression and the solution may have severe numerical stability problems. The matrix of the system is formed from inner products in a vector space, and if the linear system is solved by inverting the matrix, there will almost surely be a loss of accuracy. The preconditioning of the data is also important and is discussed.

Next, the important area of relative importance analysis is considered. The most common use of this area is to rank a set of variables in importance. The goal of this paper is different in that we determine the relative importance of a set of variables as compared to a different set.

We then state the all-important Hilbert Projection Theorem and then restate it in terms of linear regression as a special case. The numerical tools are all chosen to produce the QR factorization of a matrix. A main result is that linear regression is reduced to matrix multiplication.

Lastly, the main method of this paper is developed, using vector space methods for relative importance analysis. We use vector space methods to predict the relative importance of a set of variables.

### 5.1. Multi-Linear Regression: Background

Given a data set of the form

$$\begin{array}{cccc} X_{1} & X_{2} & \cdots & X_{M-1}\\ x_{11} & x_{12} & \cdots & x_{1,M-1}\\ \vdots & \vdots & & \vdots \\ x_{N1} & x_{N2} & \cdots & x_{N,M-1} \end{array},$$

the goal is to determine how well a separate variable of the form

$$\begin{array}{c} X_{M}\\ x_{1M}\\ x_{2M}\\ \vdots \\ x_{NM} \end{array}$$

can be approximated by the above data. A standard procedure from multiple linear regression is to construct a function of the form

$$F\left(x_{i1},x_{i2},\cdots ,x_{i,M-1}\right)=\alpha_{1}x_{i1}+\cdots +\alpha_{M-1}x_{i,M-1}+C$$

that minimizes the expression

$$L=\sum_{i=1}^{N}{\left(F\left(x_{i1},x_{i2},\cdots ,x_{i,M-1}\right)-x_{iM}\right)}^{2}.$$

The expression *L* is a function of the α’s and *C*, and the minimum is easily found by calculating the partial derivatives of *L* with respect to the unknown variables and then solving the resulting linear equation given below.   

(1) $$\begin{array}{ccc} \left(\begin{array}{cccc} \langle X_{1},X_{1}\rangle & \langle X_{1},X_{2}\rangle & \cdots & \langle X_{1},X_{M-1}\rangle \\ \langle X_{2},X_{1}\rangle & \langle X_{2},X_{2}\rangle & \cdots & \langle X_{2},X_{M-1}\rangle \\ \vdots & \vdots & & \vdots \\ \langle X_{M-1},X_{1}\rangle & \langle X_{M-1},X_{2}\rangle & \cdots & \langle X_{M-1},X_{M-1}\rangle \\ \sum_{i=1}^{N}x_{i1} & \sum_{i=1}^{N}x_{i2} & \cdots & \sum_{i=1}x_{i,M-1} \end{array}\right)\left(\begin{array}{c} \alpha_{1}\\ \alpha_{2}\\ \vdots \\ \alpha_{N}\\ C \end{array}\right) & = & \\ \left(\begin{array}{c} \langle X_{1},X_{M}\rangle \\ \langle X_{2},X_{M}\rangle \\ \vdots \\ \langle X_{M-1},X_{M}\rangle \\ 0 \end{array}\right) \end{array}$$

Here, the notation $\langle X_{i},X_{j}\rangle$ is taken to be the vector space inner product

$$\langle X_{i},X_{j}\rangle =\sum_{k=1}^{N}x_{ki}x_{kj}.$$

So, whenever possible, we solve the linear equation for the unknown variables. A major problem often arises with this approach. Solving this equation can be, and usually is, numerically unstable. That is, whereas the data used may be accurate to several decimal places, the resulting numbers of the solution may have 0 decimal place accuracy.

To prevent the loss of accuracy, we begin by transforming the data into a more useful form. It is useful to precondition the data by transforming the data set using the transformation

(2) $$T\left(x_{ij}\right)=x_{ij}-E\left[X_{j}\right]$$

on each variable where

$$E\left[X_{i}\right]=\frac{1}{N}\sum_{j=1}^{N}x_{ji}$$

denotes the average or mean value of the set $X_{i}$. The covariance is given by

$$cov\left(X_{i},X_{j}\right)=E\left[\left(X_{i}-E\left[X-i\right]\right)\left(X_{j}-E\left[X_{j}\right]\right)\right].$$

The standard notation

σ(X)

will be used for the standard deviation of the set *X*. We denote the transformed data set as

(3) $$\begin{array}{ccccc} {\hat{X}}_{1} & {\hat{X}}_{2} & \cdots & {\hat{X}}_{M-1} & {\hat{X}}_{M}\\ {\hat{x}}_{11} & {\hat{x}}_{12} & \cdots & {\hat{x}}_{1,M-1} & {\hat{x}}_{1M}\\ \vdots & \vdots & \; & \vdots & \vdots \\ {\hat{x}}_{N1} & {\hat{x}}_{N2} & \cdots & {\hat{x}}_{N,M-1} & {\hat{x}}_{NM} \end{array}.$$

This generates the system of equations

(4) $$\begin{array}{ccc} \left(\begin{array}{cccc} cov({\hat{X}}_{1},{\hat{X}}_{1}) & cov({\hat{X}}_{1},{\hat{X}}_{2}) & \cdots & cov({\hat{X}}_{1},{\hat{X}}_{M-1})\\ cov(X_{2},X_{1}) & cov({\hat{X}}_{2},{\hat{X}}_{2}) & \cdots & cov({\hat{X}}_{2},{\hat{X}}_{M-1})\\ \vdots & \vdots & & \vdots \\ cov({\hat{X}}_{M-1},{\hat{X}}_{1}) & cov({\hat{X}}_{M-1},{\hat{X}}_{2}) & \cdots & cov({\hat{X}}_{M-1},{\hat{X}}_{M-1})\\ 0 & 0 & \cdots & 0 \end{array}\right)\left(\begin{array}{c} \alpha_{1}\\ \alpha_{2}\\ \vdots \\ \alpha_{N}\\ C \end{array}\right) & = & \\ \left(\begin{array}{c} cov({\hat{X}}_{1},{\hat{X}}_{M})\\ cov({\hat{X}}_{2},{\hat{X}}_{M})\\ \vdots \\ cov({\hat{X}}_{M-1},{\hat{X}}_{M})\\ 0 \end{array}\right). \end{array}$$

Note that in this setting, C=0. There is still a serious problem here in that we are assuming there is no missing data. We will assume that all data have been transformed to obtain 0 mean (in practice $|E\left(X_{i}\right)|<10^{-15}$).

### 5.2. Vector Space Methods

The main goal of this paper is to show that vector space methods can be used to predict the relative importance of a chemical in a mixture on a health outcome. We begin by discussing the primary theorem of the field, the Hilbert Projection Theorem, and then show how to use numerical linear algebra to complete the analysis.

David Luenberger was a pioneer in vector space methods in optimization in the late 1960s. His “red book” [39] has been the standard in the field for over a half century. The basis for the subject is a vector space, either finite or infinite dimensional, that is equipped with a norm. For our purposes, here, we will use an n-dimensional space usually denoted by $R^{n}$, and the norm is defined as

$${∥x∥}^{2}=\frac{1}{n}\left(x_{1}^{2}+\cdots +x^{2}\right).$$

We include the multiplier 1/n in order to have

∥x∥=standarddeviation(x)=σ(x),

and we use the inner product

$$\langle x,y\rangle =\frac{1}{n}\sum_{i=1}^{n}x_{i}y_{i},$$

again to recover covariance.

Given a linear subspace of $R^{n}$, say

$$V=\mathrm{span}\{Y_{1},\cdots ,Y_{k}\},$$

and a vector *X* not in *V*, a classic problem is to find the vector in *V* that best approximates *X*. That is, we are searching for a vector *Y* in V of the form $Y=\alpha_{1}Y_{1}+\cdots +\alpha_{k}Y_{k}$ that minimizes ∥Y−X∥. Note that if we let

$$Y_{i}=\left(y_{1i},\cdots ,y_{ni}\right),$$

then we are trying to find

$$min_{\alpha }\frac{1}{n}\;\sum_{j=1}^{k}{\left(\alpha_{1}y_{1j}+\cdots \alpha_{n}y_{nj}-x_{j}\right)}^{2}.$$

Again, we could solve this problem by taking derivatives and solving the resulting linear equations. However, it can be solved without calculus due to a theorem commonly attributed to David Hilbert, the Hilbert Projection Theorem.

**Theorem** **1** (Hilbert Projection Theorem)**.** *For every vector x in a Hilbert space H and every nonempty closed convex set C⊂H, there exists a unique vector m∈C for which ∥x−c∥ is equal to $\inf_{c\in C}∥x-c∥.$ If the closed subset C is also a vector subspace of H, then this minimizer m is the unique element in C such that m−x is orthogonal to every member of C.*

The important part of the theorem for this paper can be restated in the following manner.

**Theorem** **2** (Regression a la Hilbert)**.** *Given a set of data vectors $\left\{Y_{1},\cdots ,Y_{m}\right\}$, let C be the set of all linear combinations of the vectors $Y_{i}$. Let X be any data vector of the same length. Then, there exists a unique Y∈C such that the*

$$\sigma \left(Y-X\right)=\underset{Z\in C}{\inf}\sigma \left(Z-X\right),$$

*and furthermore*

cov(Y−X,Z)=0

*for every Z∈C.*

Every vector in *C* is of the form

$$\alpha_{1}Y_{1}+\alpha_{2}Y_{2}+\cdots +\alpha_{m}Y_{m},$$

and by the theorem, we must have that for each $Y_{i}$

$$cov(\alpha_{1}Y_{1}+\alpha_{2}Y_{2}+\cdots +\alpha_{m}Y_{m}-X,Y_{i})=0;$$

so we obtain the equation

$$\begin{array}{ccc} \left(\begin{array}{cccc} cov(Y_{1},Y_{1}) & cov(Y_{1},Y_{2}) & \cdots & cov(Y_{1},Y_{m})\\ cov(Y_{2},Y_{1}) & cov(Y_{2},Y_{2}) & \cdots & cov(Y_{2},Y_{m})\\ \vdots & \vdots & & \vdots \\ cov(Y_{m},Y_{1}) & cov(Y_{m},Y_{2}) & \cdots & cov(Y_{m},Y_{m}) \end{array}\right)\left(\begin{array}{c} \alpha_{1}\\ \alpha_{2}\\ \vdots \\ \alpha_{m} \end{array}\right) & = & \\ \left(\begin{array}{c} cov(Y_{1},X)\\ cov(Y_{2},X)\\ \vdots \\ cov(Y_{m},X) \end{array}\right). \end{array}$$

Note that there is no guarantee that there exists a unique solution to this set of equations. The only guarantee is that that there is a unique vector that satisfies the theorem. However, it will be a rare set of data for which this set fails to have a unique set of solutions. It will fail if and only if there exists $\beta_{i}$ with $\sum_{i=1}^{m}\beta_{i}^{2}\neq 0$, and $\sum_{i=1}^{m}\beta_{i}Y_{i}=0$, i.e., the set is not linearly independent. This material is contained in [39], and there, it is credited to R. Kalman [44].

Now, since cov(Y−X,Y)=0, we have the all important formula,

$$\sigma {\left(Y-X\right)}^{2}+\sigma {\left(Y\right)}^{2}=\sigma {\left(X\right)}^{2}=1,$$

which is just the Pythagorean formula. Thus

$$\sigma \left(Y\right)=\sqrt{1-\sigma {\left(Y-X\right)}^{2}}.$$

Recall that a matrix A is orthonormal if and only if i≠j then $\langle A_{i},A_{j}\rangle =0$ and $\langle A_{i},A_{i}\rangle =1$ for columns of A, $A_{i}$.

We let the data set of Equation (3) be represented by the matrix

$$X=\left(\begin{array}{ccccc} {\hat{x}}_{11} & {\hat{x}}_{12} & \cdots & {\hat{x}}_{1,M-1} & {\hat{x}}_{1M}\\ \vdots & \vdots & & \vdots & \vdots \\ {\hat{x}}_{N1} & {\hat{x}}_{N2} & \cdots & {\hat{x}}_{N,M-1} & {\hat{x}}_{NM} \end{array}\right).$$

Typically, we will have N>>M, and we are interested in the vector space spanned by the columns of X. In fact, we are letting the vector space be the surrogate for the information contained in the data [37,38]. We are going to use the vector space rather than the particular data vectors. This is the primary difference between what we are doing here versus the work of of [29]. Our goal is to factor the matrix X as the product of an orthonormal matrix and an upper triangular matrix,

X=QR.

The columns of Q will be the basis we are seeking, and the matrix R allows us to go back to the original basis assuring us that Q and X generate the same vector space or, in other words, both represent the same set of information. Let the *i*th column of X be denoted by $X_{i}$. Our first step is to normalize the the data. Let

$$D=diag(∥X_{1}∥,∥X_{2}∥,\cdots ,∥X_{m}∥)$$

where *D* is a diagonal matrix. Replace X with $XD^{-1}$. Note that the columns of this matrix span the same vector space as the columns of X. The classical method for producing an orthonormal basis is to apply the Gram-Schmidt process to the columns of X. The algorithm is as follows.

$$\begin{array}{ccc} Q_{1} & = & X_{1}\\ {\bar{Q}}_{2} & = & X_{2}-\langle X_{2},X_{1}\rangle X_{1}\\ Q_{2} & = & {\bar{Q}}_{2}/∥{\bar{Q}}_{2}∥\\ \vdots & & \vdots \\ {\bar{Q}}_{k} & = & X_{k}-\langle X_{k},X_{1}\rangle X_{1}-\cdots -\langle X_{k},X_{k-1}\rangle X_{k-1}\\ Q_{k} & = & {\bar{Q}}_{k}/∥{\bar{Q}}_{k}∥\\ \vdots & & \vdots \end{array}$$

Note that the algorithm is just the repeated application of the Hilbert Projection Theorem. Unfortunately, the algorithm is numerically unstable (the fifth author of the paper is indebted to Professors Victoria Howle and Gregory Ammar for pointing me in the direction of stable algorithms). There are four main algorithms that are known to be numerically far superior to the classical Gram–Schmidt. They are the modified Gram–Schmidt, the Householder QR algorithm, Givens rotation, and singular value decomposition. We will use these four algorithms in our application in Section 3. See Appendix A for more information.

### 5.3. Relative Importance via Approximation

In this section, we develop the main mathematical result of the paper, which is a form of relative importance analysis using vector space methods.

Again, we begin with a data set of the form

$$\begin{array}{cccccccc} X_{1} & X_{2} & \cdots & X_{M-1} & Y_{1} & \cdots & Y_{K} & X_{M}\\ x_{11} & x_{12} & \cdots & x_{1,M-1} & y_{11} & \cdots & y_{1K} & x_{1M}\\ \vdots & \vdots & & \vdots & \vdots & & \vdots & \vdots \\ x_{N1} & x_{N2} & \cdots & x_{N,M-1} & y_{N1} & \cdots & y_{NK} & x_{NM} \end{array}$$

We repeat the procedure for the matrix *X* and factor it as X=QR and obtain the best approximation as

$$A_{X}\left(X_{M}\right)=QQ^{\prime }X_{M}.$$

The Hilbert Projection Theorem says that the vector

$$X_{M}-A_{\bar{XY}}\left(X_{M}\right)$$

is the solution of the problem

$$\min_{z\in \mathrm{span}(\bar{XY})}∥z-X_{M}∥$$

and that

$$X_{M}-A_{X}\left(X_{M}\right)$$

is the solution of

$$\min_{z\in \mathrm{span}(X)}∥z-X_{M}∥.$$

We then have the important fact that

$$∥X_{M}-A_{\bar{XY}}\left(X_{M}\right)∥\leq ∥X_{M}-A_{X}\left(X_{M}\right)∥$$

since

$$\mathrm{span}\left(X\right)\subset \mathrm{span}\left(\bar{XY}\right).$$

From this, we conclude that

$$∥A_{\bar{XY}}\left(X_{M}\right)∥\geq ∥A_{X}\left(X_{M}\right)∥.$$

The ratio of

$$\frac{∥A_{\bar{XY}}\left(X_{M}\right)∥-∥A_{X}\left(X_{M}\right)∥}{∥A_{X}\left(X_{M}\right)∥}$$

is the percentage increase due to adding the *Y* data to the *X* data set.

## 6. Conclusions

This paper demonstrates the use of vector space methods combined with numerical linear algebra to develop a tool for measuring the effect of a set of toxicants (PFAS) on a biological target (bone) relative to a given set of quantities (other variables). By “other variables”, we mean any variables that might to selected to answer a question of interest. We showed that by using a QR algorithm, the calculations were reduced to matrix multiplications, and the result had a high degree of numerical accuracy. We proposed four methods for determining the QR factorization and presented pseudo-algorithms for each. We have demonstrated that this tool can be used to evaluate relative contributions of environmental contaminants to complex diseases and conditions with multiple etiologies. We hope that this will help simplify risk assessments and cost–benefit analyses for chemicals in the environment. We further anticipate that this approach will be useful for ecological or other types of multi-factorial risk assessments.

We believe the most immediately important result of this exercise is that we have demonstrated that women are more susceptible to PFAS-influenced changes in BMD than men. We therefore support the examination and evaluation of PFAS health effects according to gender. This will be important in avoiding obscuring effects if data from both genders are pooled, and one gender (male or female) is more vulnerable.

## Figures and Tables

**Table 1:** Acronyms and Formula Abbreviations.

Abreviation	Definition
A	Age
AL	Albumin
BMD	Bone Mineral Density
D2	Vitamin D2
D3	Vitamin D3
E	Estradiol
ED3	Epi-25OHD3
FFF	Firefighting Foam
G	Gender
H	Height
Me-PFOSA-AcOH	2-(N-methyl-PFOSA) acetate
MLR	Multi-Linear Response
NHANES	National Health and Nutrition Examination Surveys
PFAS	Per and Polyfluorinated Alkyl Substances
PFDA	Perfluorodecanoic acid
PFHxS	Perfluorohexane sulfonic acid
PFNA	Perfluorononanoic acid
PFUA	Perfluoroundecanoic acid (PFUA)
n-PFOS	n-perfluorooctane sulfonic acid
P	Potassium
R	Race
SHBG	Sex Hormone Binding Globulin
T	Testosterone
W	Weight

**Table 2:** PFAS Test Percentages.

PFAS	NHANES Code	N	Below	Percentage above
PFDA	LBXPFDE	2168	455	79%
PFHxS	LBXFHS	2168	25	99%
Me-PFOSA-AcOH	LBXMe-PFOSA-AcOH	2168	1204	44%
PFBS	LBXPFBS	2168	2153	.6%
PFUnA	LBXPFHP	2168	1897	12%
PFNA	LBXPFNA	2168	27	99%
PFUA	LBXPFUA	2168	1225	43%
Branched PFOA isomers	LBXPFDO	2168	1801	17%
PFOS	LBXNFOS	1918	11	99%

**Table 3:** Variables Nomenclature.

Name	Abreviation	NHANES Code
Age	A	RIDAGEYR
Albumin	AL	URXUMS
Weight	W	BMXWT
Height	H	BMIHT
Testostrone	T	LBXTST
Estridol	E	LBXEST
Vitamin D2	D2	LBXVD2Ms
Vitamin D3	D3	LBXVD3MS
Epi-250HD3	ED3	LBXVE3MS
Gender	G	RIAGENDR
Race	R	RIDRETH1
SHBG	S	LBXSHBG
Potassium	P	LBXSKSI
Trunk Bone Mineral Density	TBMD	DXDTRBMD

**Table 4:** PFAS Nomenclature.

Chemical	PFAS	NHANES Code
Perfluorodecanoic acid	PFDA	LBXPFDE
Perfluorohexane sulfonic acid	PFHxS	LBXPFHS
2-(N-methyl-PFOSA) acetate	Me-PFOSA-AcOH	LBXMPAH
Perfluorononanoic acid	PFNA	LBXPFNA
Perfluoroundecanoic acid	PFUnA	LBXPFUA
n-perfluorooctane sulfonic acid	n-PFOS	LBXMFOS

**Table 5:** Approximation Results.

	With PFAS	Without PFAS	Percent Increase
Combined	0.933	0.901	4.7%
Female	0.639	0.585	11.1%
Male	0.805	0.787	3.2%

## Data Availability

All data used in this analysis are freely available from the US Centers for Disease Control. https://www.cdc.gov/nchs/nhanes/index.htm (accessed on 4 November 2022).

## Appendix A

There are five main algorithms (Algorithms A1–A5) that are known to be far superior numerically to the classical Gram–Schmidt. The first is the modified Gram–Schmidt. It is very fast on moderate sized matrices and very accurate. See [41] for details on accuracy.

The second is the Householder QR algorithm. It gave very good results on matrices up to 600 by 30. It was not used on larger matrices for this paper.

The third algorithm is the Givens rotation for computing the QR factorization. It was noticeably slow on matrices of size 600 by 30, but the accuracy was the same as the Householder algorithm.

The fourth algorithm is the classical singular valued factorization algorithm. It is preferred by many.

We give here the pseudo codes for these five algorithms (Algorithms A1–A5) below. For a complete analysis of the five factorization algorithms, see [42].

**Algorithm A1** Modified Gram–Schmidt QR Factorization
**Input: $X_{m,n}$** **Output: $Q_{m,n},R_{n,n}$** 1: **procedure** GRAM-SCHMIDT(X) 2: [d,n]=size(X) 3: m=min(d,n) 4: R=zeros(m,n) 5: Q=zeros(d,m) 6: **for** i = 1:m **do** 7: v=X(:,i) 8: **for** j = 1:i-1 **do** 9: $R_{i,j}=Q_{:,j}^{⊺}$ 10: $v=v-R_{j,i}*Q_{:,j}$ 11: **end for** 12: $R_{i,i}=norm\left(v\right)$ 13: $Q_{:,i}=\frac{v}{R_{i,i}}$ 14: **end for** 15: $R_{:,m+1:n}\leftarrow Q^{⊺}*X_{:,m+1:n}$ 16: **end procedure**

**Algorithm A2** Householder QR Factorization
**Input: $X_{m,n}$** **Output: $Q_{m,n},R_{n,n}$** 1: **procedure** HOUSEHOLDER(X) 2: [m,n]=size(X) 3: $Q=I_{n}$ 4: R=X 5: **for** i = 1:n **do** 6: $e1=R_{i,i}+sign\left(R\left(i,i\right)\right)*||R_{i:,i}\left|\right|$ 7: $v=\frac{R_{i:,i}}{e1}$ 8: $\alpha =\frac{sign(R_{i,i})*e1}{\left|\right|R_{i:,i}\left|\right|}$ 9: $R_{i:,i}=R_{i:,i}-\left(\alpha *v\right)*\left(v^{⊺}*R_{i:,:}\right)$ 10: $Q_{:,i:end}=Q_{:,i:end}-\left(Q_{:,i:end}*v\right)*{\left(alpha*v\right)}^{⊺}$ 11: **end for** 12: $R=R_{1:n,1:n}$ ▷ reduce R to an n,n matrix 13: $Q=Q_{1:m,1:n}$ ▷ reduce Q to an m,n matrix 14: **end procedure**

**Algorithm A3** Givens Rotation QR Factorization
**Input: $X_{m,n}$** **Output: $Q_{m,n},R_{n,n}$** 1: **procedure** GIVENS-ROTATION(X) 2: [m,n]=size(X) 3: $Q=I_{n}$ 4: R=X 5: **for** i = 1:n **do** 6: **for** j = m:-1:i+1 **do** 7: $G=I_{m}$ 8: $\left[c,s\right]=rotate\left(R_{j=1,i},R_{j,i}\right)$ 9: G([j−1,j],[j−1,j])=[c−s;sc] 10: $R=G^{⊺}*R$ 11: Q=Q∗G 12: **end for** 13: **end for** 14: $R=R_{1:n,1:n}$ ▷ reduce R to an n,n matrix 15: $Q=Q_{1:m,1:n}$ ▷ reduce Q to an m,n matrix 16: **end procedure**

**Algorithm A4** Singular Value Decomposition
**Input: $X_{m,n}$** **Output: U,S,V** 1: **procedure** SVD(X) 2: [m,n]=size(X) 3: $U,V=I_{m}$ 4: stop=max(abs(X))∗1.e−15; 5: Xr=inf 6: **while** Xr>stop **do** 7: Q,R=householder(X) 8: U=U∗Q 9: $Q,R=householder(R^{⊺})$ 10: V=V∗Q 11: $X=R^{⊺}$ 12: Xr=||tril(X,−1)|| 13: **end while** 14: $U=U_{:,1:n}$ 15: $S=triu(X_{1:m,1:n})$ 16: **end procedure**

**Algorithm A5** Modified Bootstrap
**Input: $X_{m,n},o,p$** **Output: $J_{m,2}$** 1: **procedure** BOOTSTRAP(X, o, p) 2: [a,b]=size(X) 3: n=0 4: **while** n < a-2 **do** 5: n++ 6: kn=random(1,a) 7: T=deleterowknandnfromX 8: $T_{i}=\frac{\left(T_{ij}-E\left(T_{i}\right)\right)}{\left|\right|T_{i}\left|\right|}$ 9: A=T(:,1:o) 10: B=T(:,1:o+p) 11: C=T(:,end) 12: [QA,RA]=householderQR(A) 13: [QB,RB]=householderQR(B) 14: $D=QA*QA^{⊺}*C$ 15: $E=QB*QB^{⊺}*C$ 16: J+=[||D||,||E||] 17: **end while** 18: return *J* 19: **end procedure**
